# Correction: Influence of *Funneliformismosseae* enhanced with titanium dioxide nanoparticles (TiO_2_NPs) on *Phaseolus vulgaris* L. under salinity stress

**DOI:** 10.1371/journal.pone.0243491

**Published:** 2020-12-03

**Authors:** Nashwa El-Gazzar, Khalid Almaary, Ahmed Ismail, Giancarlo Polizzi

There are errors in the Funding statement. The correct Funding statement is as follows: This work was supported by the Researchers supporting Project (RSP-2020/189) King Saud University, Riyadh, Saudi Arabia. The funders had no role in study design, data collection and analysis, decision to publish, or preparation of the manuscript.

There is an error in affiliation 4 for author Giancarlo Polizzi. The correct affiliation 4 is: Dipartimento di Agricoltura, Alimentazione e Ambiente, Sezione di Patologiavegetale, University of Catania, Italy.

## References

[1] El-Gazzar N, Almaary K, Ismail A, Polizzi G (2020) Influence of *Funneliformismosseae* enhanced with titanium dioxide nanoparticles (TiO_2_NPs) on *Phaseolus vulgaris* L. under salinity stress. PLoS ONE 15(8): e0235355 10.1371/journal.pone.0235355 32817671 PMC7446817

