# Supplementary material for: Influence of Funneliformis mosseae enhanced with titanium dioxide nanoparticles (TiO2NPs) on Phaseolus vulgaris L. under salinity stress
Source: PLoS One. 2020 Aug 20;15(8):e0235355. doi: 10.1371/journal.pone.0235355 (PMC7446817; doi:10.1371/journal.pone.0235355)
Supplement: S1 Table — (DOCX) [file pone.0235355.s001.docx]

| Treatments | | Mean of Na (mg/g dry weight plant) | | | Mean of Ca^2+^content (mg/g Dwt.) | | | Mean of Mg^2+^ content (mg/g Dwt.) | | |
| --- | --- | --- | --- | --- | --- | --- | --- | --- | --- | --- |
|  |  |  | Shoot | T.F. | Root | Shoot | T.F. | Root | Shoot | T.F. |
| P Control | NM | 9.3h | 9.2fg | 0.98 | 19.5ab | 12.2ab | 0.62 | 8.1b | 10.6ab | 1.3 |
|  | M | 10.6h | 8.5. h | 0.80 | 22.0a | 13.1a | 0.60 | 9.9a | 14.0a | 1.4 |
| Tio_2_NPs | NM | 11.3ef | 9.5fg | 0.84 | 19.3bc | 9.4c | 0.48 | 8.5b | 11.1bc | 1.3 |
|  | M | 16.4ef | 11.2g | 0.68 | 18.2bc | 11.2b | 0.61 | 10.2b | 18.4a | 1.8 |
| NaCl 100mM | NM | 23.6e | 20.5bc | 0.86 | 17.9cd | 7.8cd | 0.43 | 6.8c | 7.1de | 1.0 |
|  | M | 28.4de | 16.2de | 0.57 | 18.7bc | 10.9c | 0.58 | 8.7b | 13.9a | 1.6 |
| NaCl 200mM | NM | 31.2d | 28.9a | 0.92 | 15.4bc | 5.2d | 0.34 | 4.9c | 4.2e | 0.85 |
|  | M | 39.5c | 18.6ab | 0.46 | 17.2cd | 9.7c | 0.56 | 6.8c | 10.1 | 1.48 |
| Tio_2_NPs+NaCl100mM | NM | 30.0c | 20.4ef | 0.68 | 17.2cd | 9.4c | 0.52 | 6.9c | 8.9 cd | 1.2 |
|  | M | 46.2a | 12.3fg | 0.27 | 16.9bc | 10.4c | 0.62 | 8.9b | 14.8cd | 1.66 |
| Tio_2_NPs+NaCl200mM | NM | 45.7b | 28.0cd | 0.61 | 12.3d | 4.3d | 0.35 | 3.6d | 3.3e | 0.9 |
|  | M | 59.0a | 13.2ef | 0.22 | 10.9cd | 6.9cd | 0.63 | 5.6c | 8.7de | 1.55 |
| L.S.D | | 5.2 | 3.3 |  | 2.7 | 1.6 |  | 1.1 | 1.8 |  |

**Supplementary Table 1.** Effect of salinity levels on sodium (Na), calcium (Ca), magnesium (Mg) and translocation.

**Factors (T F)** of mycorrhizal (M), and non-mycorrhizal plants (NM) with and without TiO_2_NPs

Translocation Factors (T F) = level of element in root / level of element in root LSD: At significant level (P> 0.05).Sample symbols (a.a) mean non significant difference (a.b) mean significant difference
