# Supplementary material for: Influence of Funneliformis mosseae enhanced with titanium dioxide nanoparticles (TiO2NPs) on Phaseolus vulgaris L. under salinity stress
Source: PLoS One. 2020 Aug 20;15(8):e0235355. doi: 10.1371/journal.pone.0235355 (PMC7446817; doi:10.1371/journal.pone.0235355)
Supplement: S2 Table — (DOCX) [file pone.0235355.s002.docx]

**Supplementary Table 2 .** Amplified Nest and chitin synthase Quantity (Intensity & Relative density) of mycorrhiza levels of Phaseolus plants inoculated with ***Funneliformis mosseae*** and TiO_2_NPs in the presence of salinity.

|  | Analysis | | | |
| --- | --- | --- | --- | --- |
|  | Amplified Nest Quantity | | Amplified Chitin synthase Quantity | |
| Treatments | Molecular ratio (quantity fold) of nest gene intensity | Molecular relative density of nest gene % | Molecular ratio (quantity fold) of Chitin synthase gene intensity | Molecular relative density of Chitin synthase gene % |
| M (Mycorrhiza) | 1.000bc | 20.000 c | 1.000bc | 20.000 c |
|  |  |  |  |  |
| M (Mycorrhiza)+ N (Tio2NPs) | 1.900 a | 52.000 a | 2.400 a | 67.000 f |
| M (Mycorrhiza) + Salinity (S 100mM) | 0.700 cd | 12.000 d | 0.700 cd | 12.000 d |
| M + N + (S100mM) | 1.200 b | 28.000 b | 1.300 b | 33.000 a |
| M + (S 200mM) | 0.500 d | 8.000 d | 0.600 d | 8.000 e |
| M+ N + (S 200mM) | 1.000bc | 20.000 c | 1.200 b | 23.000 b |
| LSD _P ≤ 0.05_ | 0.446 | 5.034 | 0.395 | 0.840 |

M = mycorrhiza N = TiO_2_NPs S = salinity, **Highly significant. LSD: At significant level (P> 0.05).Sample symbols (a.a) mean non significant difference (a.b) mean significant difference.
