# Supplementary material for: Influence of Funneliformis mosseae enhanced with titanium dioxide nanoparticles (TiO2NPs) on Phaseolus vulgaris L. under salinity stress
Source: PLoS One. 2020 Aug 20;15(8):e0235355. doi: 10.1371/journal.pone.0235355 (PMC7446817; doi:10.1371/journal.pone.0235355)
Supplement: S1 File — Brief methodologies protocol for some measurements. (PDF) [file pone.0235355.s003.pdf]

Brief methodologies protocol for some measurements

A).Mycorrhizal measurements

1-Determination of mycorrhizal root colonization.

Root samples were collected at an interval of 10 days, AM colonization of root was examined after staining with 0.5% trypan blue in lactophenolPhillips and Hayman, (1970) method. Root segments, each approximately 1cm long, were selected at random from a stained sample and mounted on microscopic slides. Slides with stained root segments were carefully observed using microscope under suitable magnification. PercentMycorrhizal root colonization (Frequency of mycorrhizal infection F%, Mycorrhizal intensity M% and Arbuscular activity A%) was determined by gridline intersect slide method (Giovannetti and Mosse, 1980).

-Determination of mycorrhizal dependency: The mycorrhizal dependency (MD) of plants was calculated according to Gerdemann, (1975) according to the following:

MD= dry wt of mycorrhizal treatments x100

drywt of non-mycorrhizal treatments

B).Plant analysis.

1- Morphological parameters

.Determination thenumber of leaves, determination of Shoot, Root length and plant height after dipping the roots in water to remove soil particles and washing with distilled water, determination of Fresh weight and Dry weight after drying at 70^{0}C for 48 hr, determination of Root to shoot ratio(R/S)through using plant root: shoot length ratios

2- Physiological parameters:

2.1. Estimation of nutrients elements.

- Estimation of inorganic phosphorus (Pi):

This was carried out according to the method modified by (El-Ayouty ,1986):

- Reagents:

Phenolphthalein indicator solution.

Standard sulfuric acid (0.025 N).

Ammonium molybdate solution (strong acid solution): 25 g $(NH4)_6Mo70_24.4H20$ were dissolved in 175 ml distilled water. 310 ml cone. H_2S0_4 were added continuously to 400 ml distilled water, after cooling, the molybdate solution was added and the solution was diluted to 1 liter.

1. Stannous chloride solution: 2.5 g of a fresh supply of SnCI2.H2O were dissolved in 10 ml conc. HC1; then diluted to 100 ml with distilled water. If turbid, filtration was done (store in cool temperature).
2. Stock phosphate solution: 0.7164 g of KH_2P0_4 (which has been dried in an oven, very well) was dissolved in distilled water. The solution was diluted to 1 liter so as 1 ml of stock solution contains 0.5 mg of $P0_4^{3"}$.

6. Standard phosphate solution: 100 ml of stock phosphate solution was diluted to 1 litter, with distilled water. 1 ml of this standard solution contains 0.05 mg of $P0_4^{3"}$.

•**Procedure:**

1. Five ml of the sample were taken and one drop of phenolphthalein indicator was added.

2-If a pink color appears, standard sulfuric acid (0.2 N) was added drop by drop until the color disappears.

3-0.2 ml of ammonium molybdate solution was added with thorough mixing after each addition.

4. Then, 0.025 ml of stannous chloride solution was added with thorough mixing.

5. After 10 minutes, the absorbance of sample was measured at 690 nm in the spectrophotometer using the reagent blank as the reference solution. Determine concentration of phosphate in the sample from a standard phosphate calibration curve.

- **Standard curve for phosphate:**

Different volumes (1 to 10 ml) of standard phosphate solution were taken in different beakers and diluted (each one) to 50 ml with distilled water. Each ml of standard $P0_4^{3"}$ solution contains 0.05 mg of $P0_4^{3}$- so it gives a range of 0.05 to 0.5mg of $PO_4^{3"}$.

The previous steps (for sample) was repeated using the applied reagents as a blank, then a curve between measured optical densities and known amounts of $P0_4$" in mg was drown.

-Estimation of Potassium element (K):

A known dry weight of plant mixed with 0.1g digestive mixture (Cole and Parkers, 1946). It consists of potassium sulphate: copper sulphate: selenium at a ratio by weight (10:1:0.5, respectively) with 2 ml concentrated sulphuric acid and Hcl, the samples were digested until the formation of clear liquid free from black residues. The digested samples were left to cool and each sample was completed to 20 ml by distilled water, the samples were measured in flame spectrophotometer.

-Estimation of nitrogen fraction:

I. Total kjeldahl-nitrogen (T.N.):

Thisfraction estimated bythe micro-Kjeldahl method according to (Allen, 1953).

- **Reagents**:

(1) concentratedsulfuric acid.

(2) Digestive mixture (Cole and Parkers, 1946). It consists of potassium sulphate: copper sulphate: selenium at a ratio by weight (10:1:0.5, respectively).

(3) Mixed indicator: 8 ml of bromocresol green (0.1 w/v) in 95% ethyl alcohol and 1 ml of methyl red (0.1 w/v).

(4)Sodium hydroxide 50% w/v.

(5)Boric acid 4%.

- **Procedure**:

A known dry weight of plant samples was transferred to 50 ml Kjeldahl flask and mixed with 2 ml concentrated sulfuric acid and 0.1 g of digestive mixture . The samples were digested until the formation of clear liquid free from black residues. The digested samples were left to cool and

each sample was completed to 20 ml by distilled water, and then transferred to the distillation apparatus. 10 ml of sodium hydroxide (50%) was added *via* the stoppered funnel, reception of ammonia was achieved by 10 ml of 4% boric acid and mixed indicator till final volume of 50 ml. Titration was carried out by N/70 hydrochloric acid and the total nitrogen in mg/g dry weight was determined: 1ml HC1 (N/70) = 0.2 mg N

II. Protein contents:

Extraction:

It was performed according to the method ofLobban*et al*.,(1988) a known plant dry weight was homogenized (in homogenizer); with little sand; in 3 ml of 0.3 M phosphate buffer (pH 6.8-7.0) until a fine tissue-water slurry was produced. Then distilled water was added up to a known volume. The cells extract was centrifuged at high speed (RCF) (X9rpm) 9-1.118x10^{-5} RS^2 for 5 minutes, then supernatant (cells extract) was decanted, and finally a known total volume was made.

Estimation:

Protein estimation was carried out according to the method of Lowery*et al*.,(1951) as follows:

- Reagents:

1- Alkaline sodium carbonate solution: 20 g Na_2C03 dissolved in 1 literof 0.1 molNaOH solution (4 g/1).

2-Copper sulphate-sodium potassium tartarate solution:

5g Cu $SO4.5H_2O$ dissolved in potassium sodium tartarate (l0g /l).

3-Alkaline solution was prepared by mixing 50 ml of reagent No.l + 1 ml of reagent No.2.

4-Folin-Ciocalteau reagent: available commercially. Folin reagent diluted with an equal volume of water on the day of use.

- **Procedure:**

Five ml of alkaline solution was added to 1 ml of the extract. After 10 min, 0.5 ml of diluted folin reagent was added, left to stand 20 min, then the optical density was determined at 750 nm.

A standard curve of bovine albumin solution in concentration of 100-1000 Ug/ml or (0.1- 0.9 mg/ml) was plotted.standard curve was constracted through dissolving 0.1 gm of bovine albumin in 100 ml dist H_2O. Then 1ml =1000Ug. Different volumes (10 to 90 ml) of standard bovine albumin solution were taken in different beakers and diluted with distilled water from (90 to 10 ml). Each 10ml of standard bovine albumin solution contains 100 ug or 0.1mg of bovine albumin so it gives a range of 0.1 to 0.9 mg of bovine albumin.

The previous steps (for sample) was repeated using the applied reagents as a blank, then a curve between measured optical densities and known amounts of bovine albumin in mg was drown.

-Estimation of enzymes acid & alkaline phosphatase.

The enzyme assay was based on the hydrolyse of substrate and estimation of the liberated inorganic phosphorus. The extraction and assay of phosphorus were carried out according to (Weimberg ,1975).

Extraction:A known fresh weight of plant tissue was ground with a volume of cold buffer approximately 5 times the weight of plant cell in homogenizer (home made). The composition of the buffer consists of 0.1 M tris-Hcl (pH 8), 0.01 M Na2-EDTA (pH 8), and 0.1% B-mercoptoethanole. The particulate matter in the extract was removed by centrifugation at 4000 rpm for 10 minutes and the supernatant was used for enzyme assay. Protein content in the supernatant was determined by Folin-phenol reagent as previously described. Quantitative estimation of enzyme activities:

For acid phosphatase: reaction mixture contains 0.4 ml of 1M acetate buffer (pH 5.5), 0.2 ml 0f 0.05M sodium pyrophosphate (pH 5.5),0.2 ml of crude extract, and 0.2 ml of dist.H2O.

For alkaline phosphatase. Reaction mixture contains 0.4 ml of 1 M biocine pH(8), 0.2 ml of 0.05 M sodium pyrophosphate pH(8),0.2 ml of 0.1 M MgCl2 and 0.2 ml of crude extract.

All the reaction mixture was incubated at 30 C for 20 minutes. Enzymatic hydrolysis was stopped by addition of 1 ml of 10% TCA. The precipitate was removed by centrifugation, and

inorganic phosphorus was measured in the supernatant using a standard curve of inorganic phosphorus. Enzyme activity expressed as Mg Pi/mg protein/ min.

-Estimation of catalase and peroxidase enzymes

• Extraction:

For the assay of catalase and peroxidase, they extracted and assayed following the method of Chance and maehly, (1955) with slight modifications. 0.1 g of fresh plant weight was homogenized in cold phosphate buffer (0.05 M at pH 6.5) in homogenizer (home-made). The homogenate was centrifuged at 10,000 rpm for 10 minutes. The pigments were removed from the supernatant by adsorbing with activated charcoal and filtered. The filtrate was completed to atotal known volume and used as an enzyme source.

(a) Assayof catalase activity:

Five ml of assay mixture for the catalase activity comprised 300 uM of Phosphate buffer (pH 6.8), 100 uM of H_20_2 and 1 ml of the enzyme extract was used. After incubation at 25°C for five minute, the reaction was stopped with the addition of 10 ml of 2% H_2S0_4 (v/v) and the residual H_20_2a was titrated against 0.01 N $Kmno_4$

(b) Assay of peroxidase activity:

According to Racusen and Foote,(1965); five ml of the assay mixture containing 300 uM of phosphate buffer (pH 6.8), 50 uM catechol, 50 uM H_20_2 and 1 ml of crude enzyme extract were prepared. After incubation at 25°C for 5 minutes, the reaction was stopped by the addition of 1ml of 10% H_2S0_4. The color was read at 430 nm and the enzyme activity was expressed as the change in the optical density/mg protein/h.
