# Supplementary material for: Influence of Funneliformis mosseae enhanced with titanium dioxide nanoparticles (TiO2NPs) on Phaseolus vulgaris L. under salinity stress
Source: PLoS One. 2020 Aug 20;15(8):e0235355. doi: 10.1371/journal.pone.0235355 (PMC7446817; doi:10.1371/journal.pone.0235355)
Supplement: S1 Raw images — (PDF) [file pone.0235355.s004.pdf]

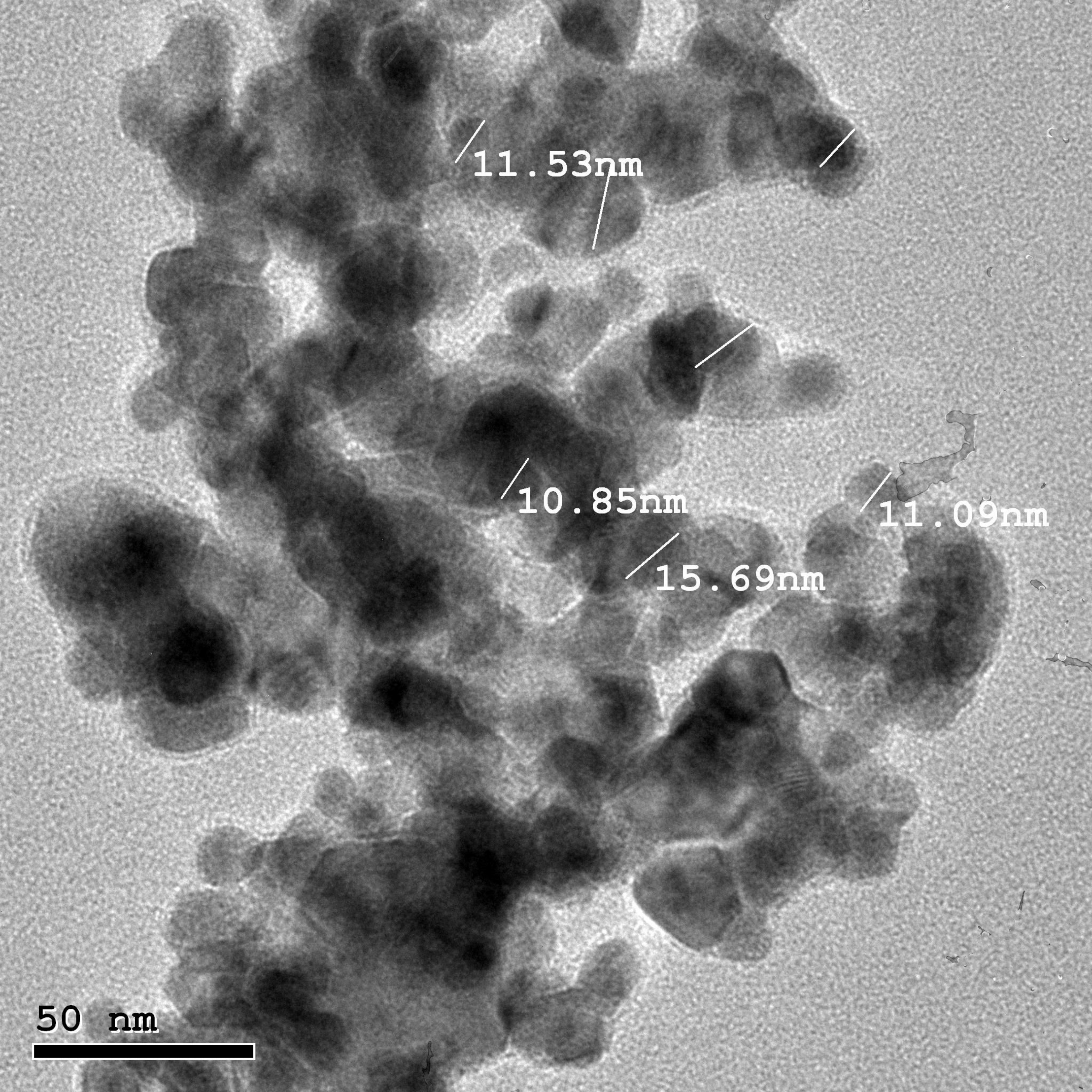
11.53nm
10.85nm
11.09nm
15.69nm
50 nm

X Number	Record 93: 4, Nashwa 3
0.4	0
0.463	0
0.536	0
0.621	0
0.719	0
0.833	0
0.965	0
1.117	0
1.294	0
1.499	0
1.736	0
2.01	0
2.328	0
2.696	0
3.122	0
3.615	0
4.187	0
4.849	0
5.615	0
6.503	0
7.531	0
8.721	0
10.1	0
11.696	0
13.545	0
15.686	0
18.166	0
21.037	0
24.363	0
28.214	0
32.674	0
37.84	0
43.821	0
50.748	11.903
58.771	33.19
68.061	33.868
78.82	16.117
91.28	3.877
105.709	0.34
122.42	0
141.772	0
164.183	0
190.137	0
220.194	0.005
255.002	0.055
295.312	0.159

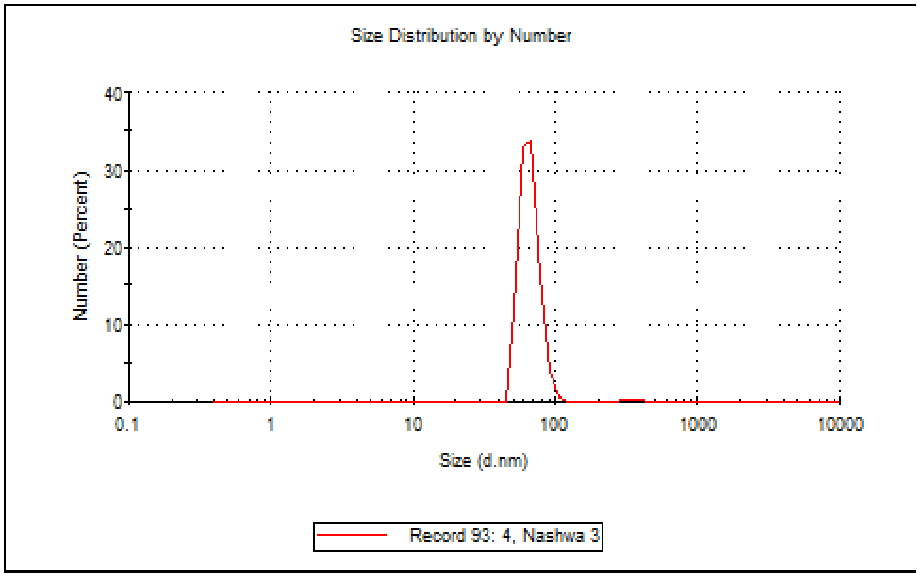

341.995	0.219
396.058	0.173
458.666	0.079
531.172	0.017
615.139	0
712.379	0
824.992	0
955.406	0
1106.435	0
1281.34	0
1483.893	0
1718.466	0
1990.119	0
2304.716	0
2669.043	0
3090.964	0
3579.581	0
4145.438	0
4800.746	0
5559.644	0
6438.508	0
7456.302	0
8634.988	0
10000	0

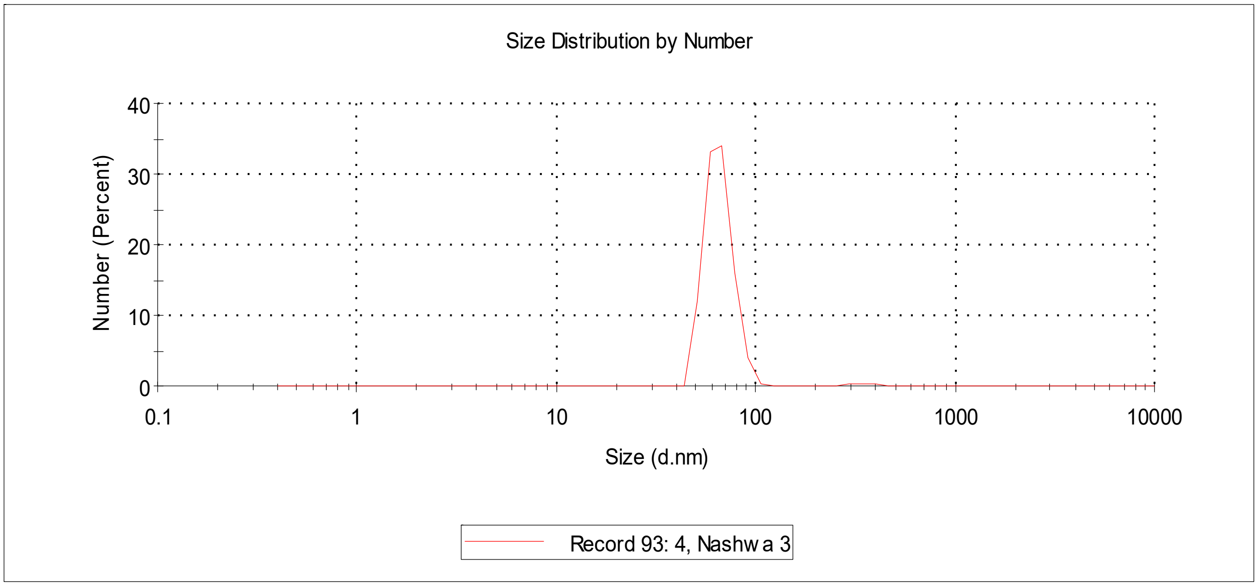
Size Distribution by Number
Number (Percent)
40
30
20
10
0
0.1
1
10
100
1000
10000
Size (d.nm)
Record 93: 4, Nashw a 3

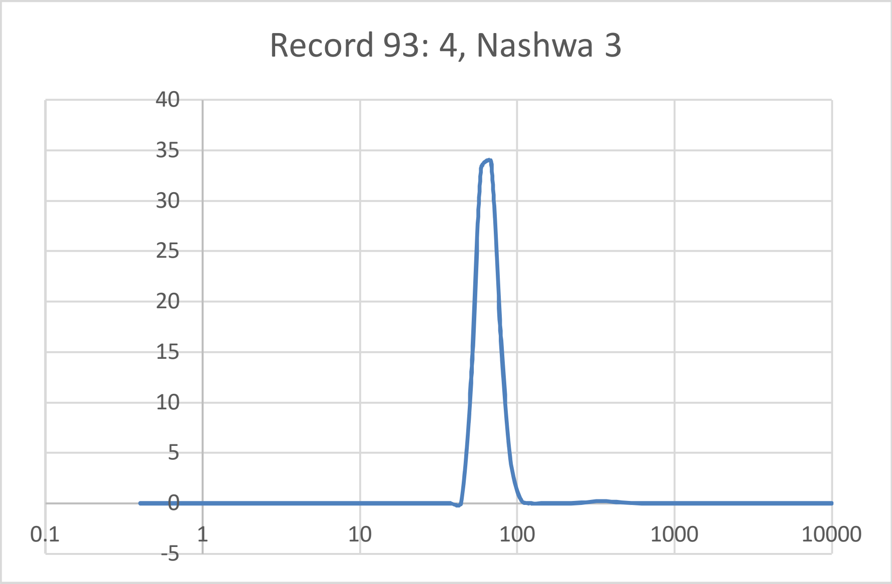
Record 93: 4, Nashwa 3
40
35
30
25
20
15
10
5
0
-5
0.1
1
10
100
1000
10000

Peak Find - Memory-68

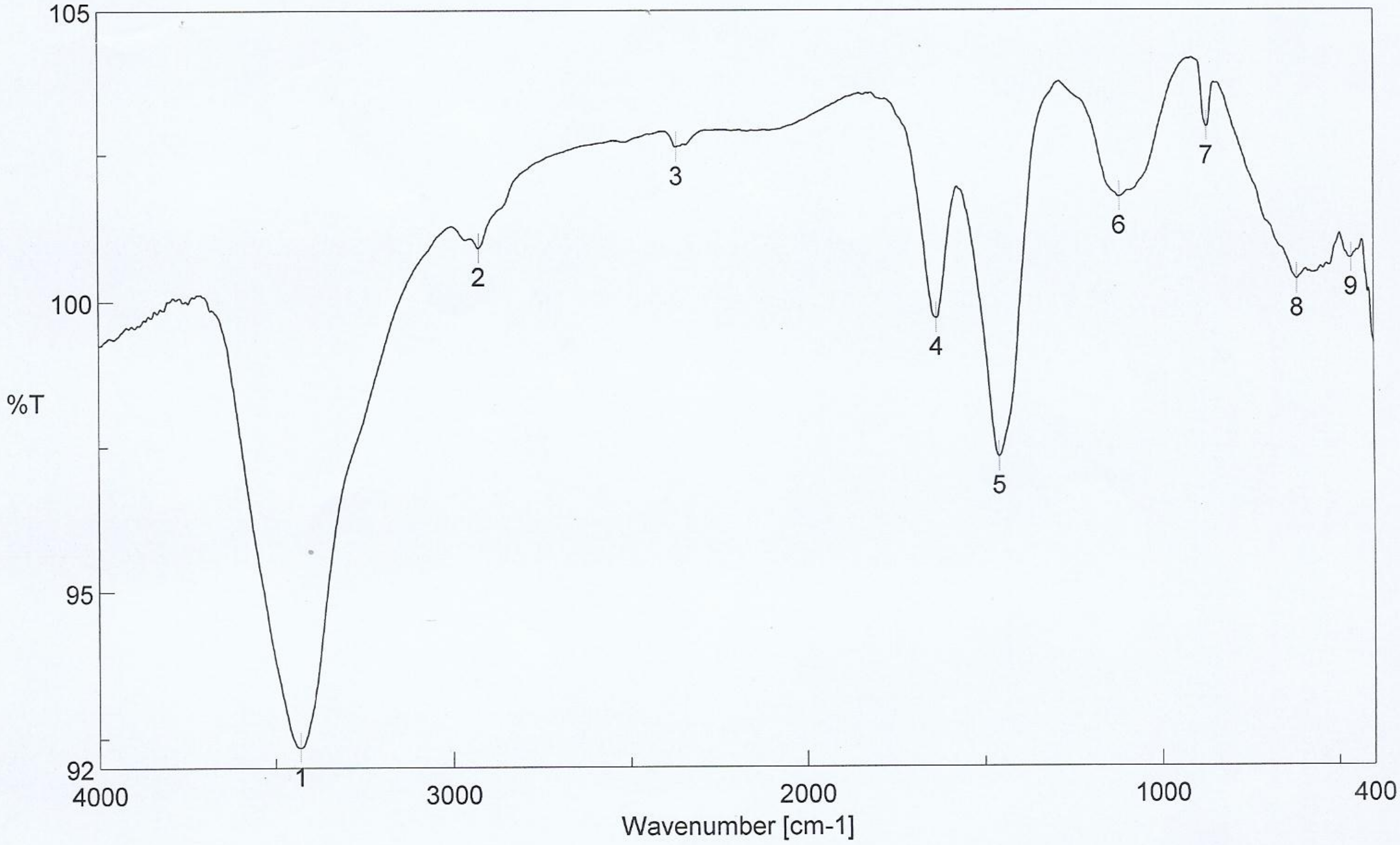

[Comments]

Sample name	2
Comment	2/2019
User	IR
Division	IR
Company	MAC

[Result of Peak Picking]

No.	Position	Intensity	No.	Position	Intensity	No.	Position	Intensity
1	3430.74	92.3423	2	2925.48	100.909	3	2366.23	102.631
4	1633.41	99.7074	5	1458.89	97.3174	6	1118.51	101.781
7	870.703	102.977	8	615.181	100.371	9	462.832	100.732

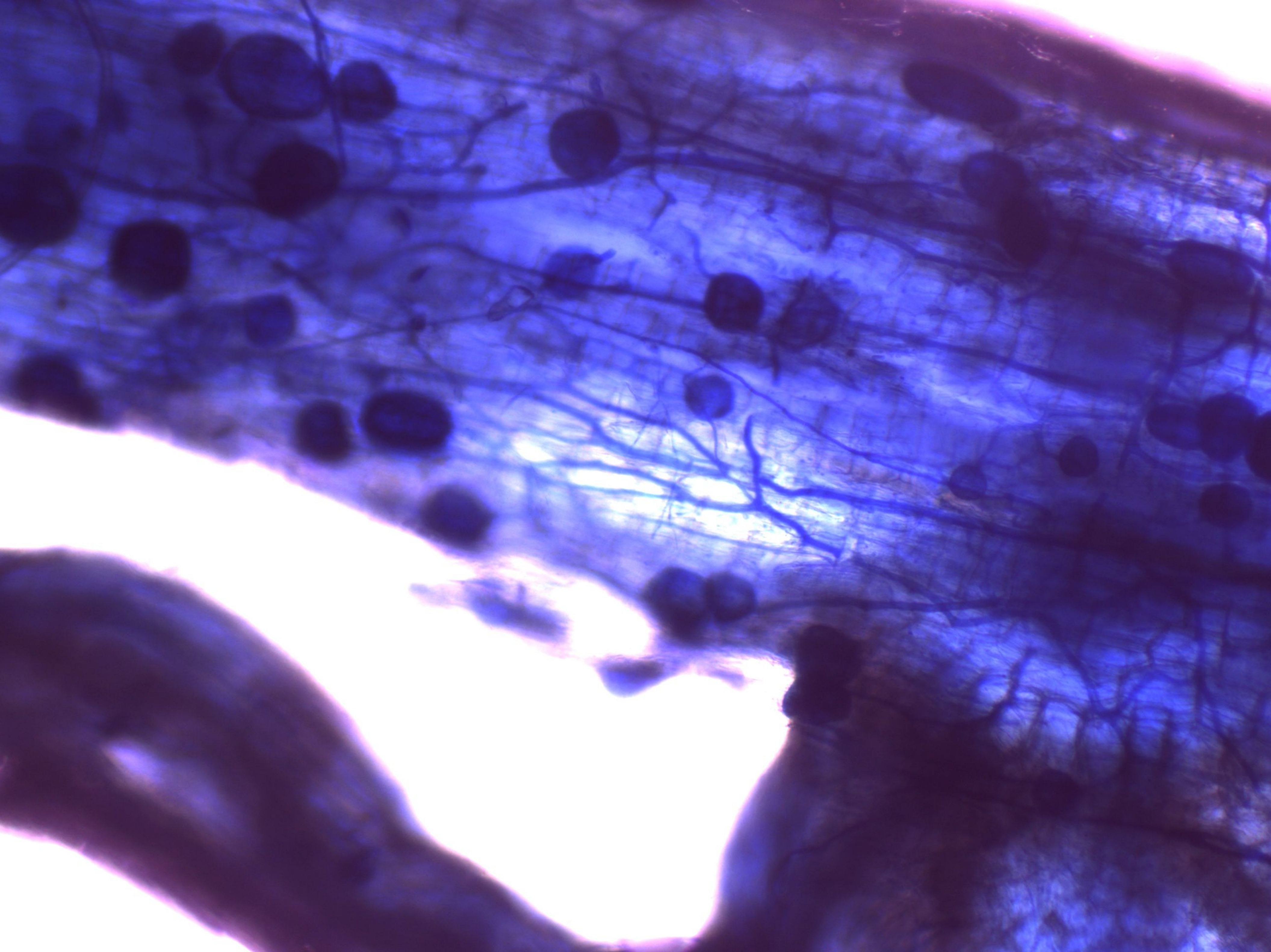

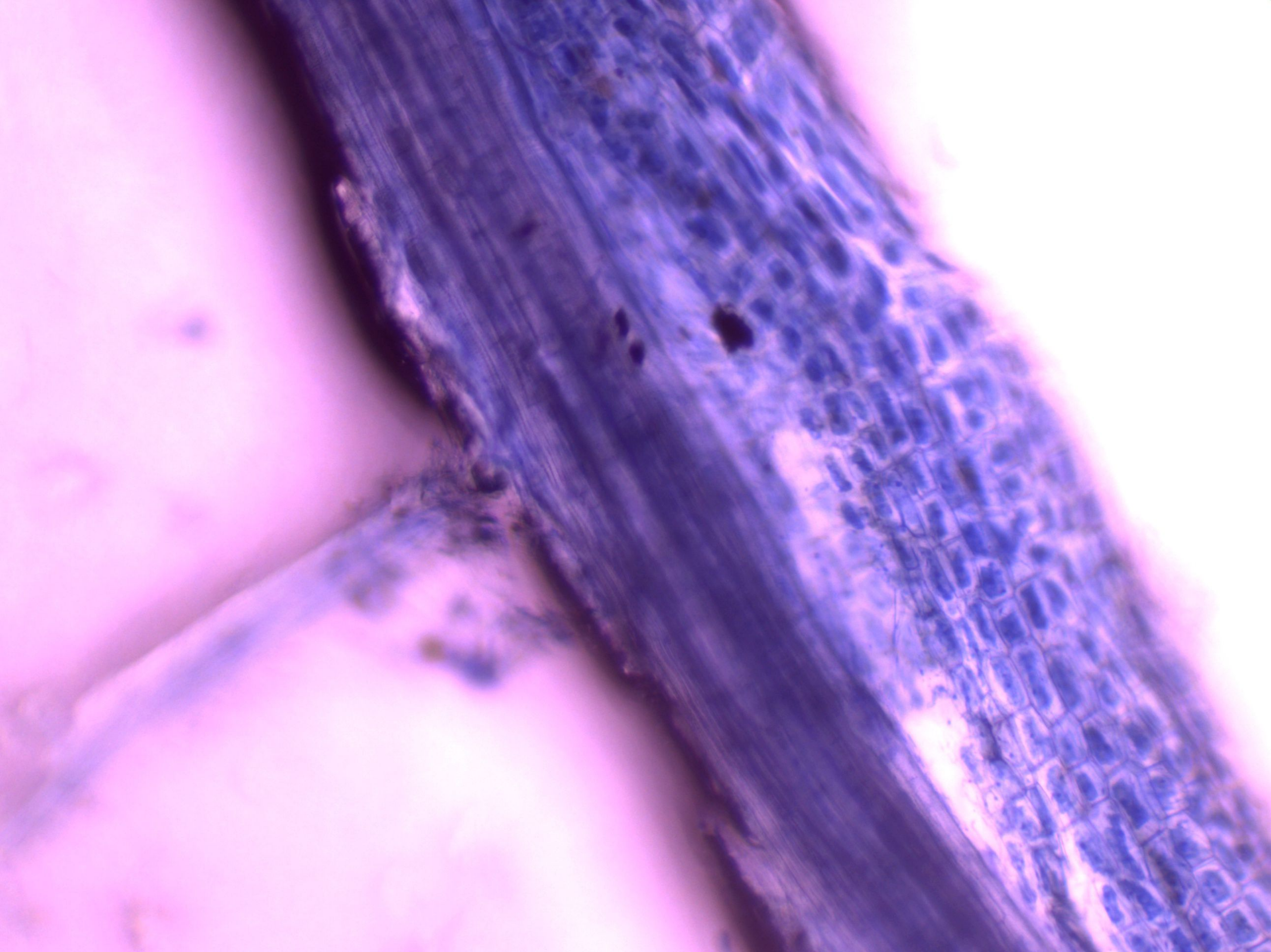

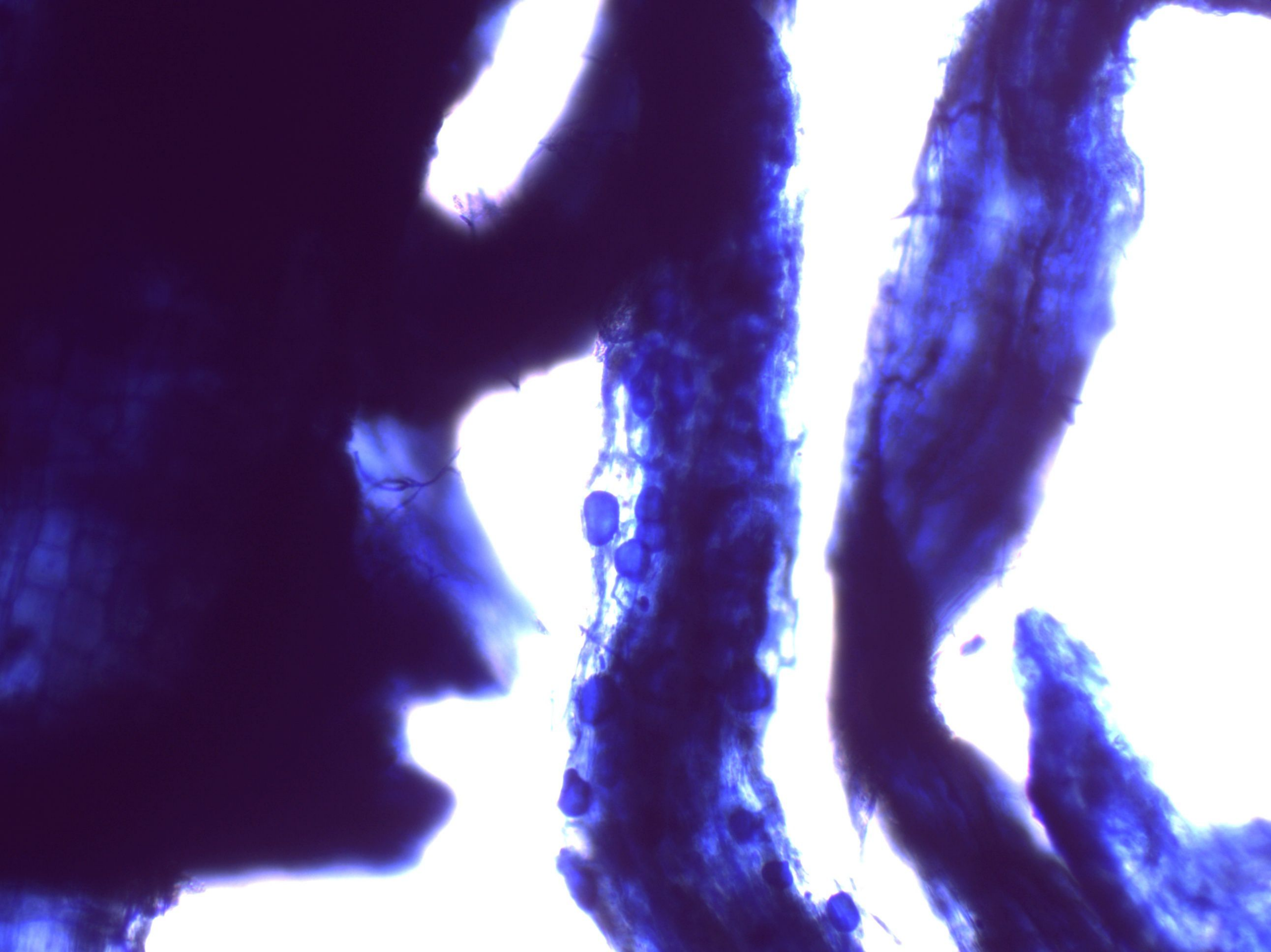

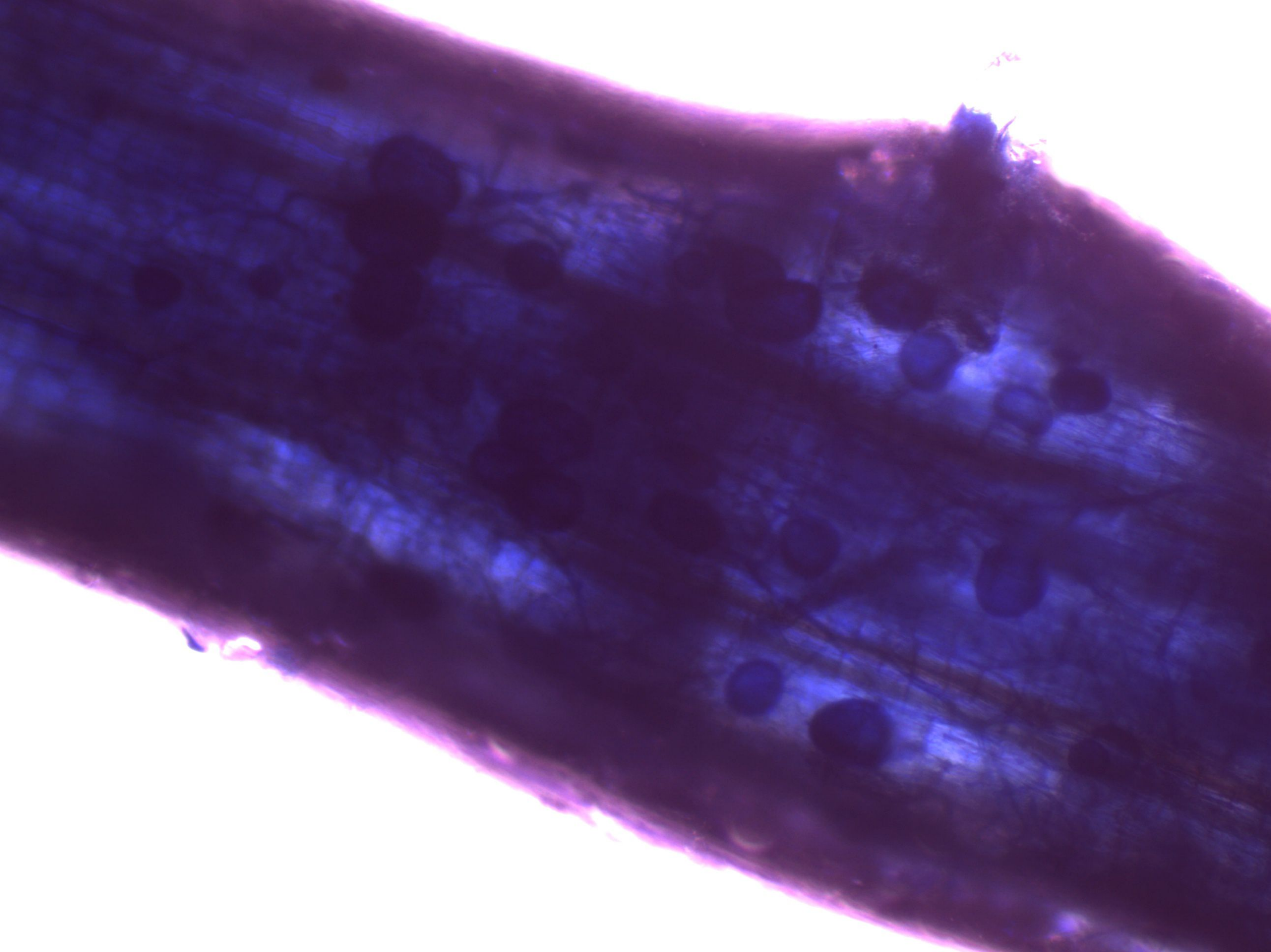

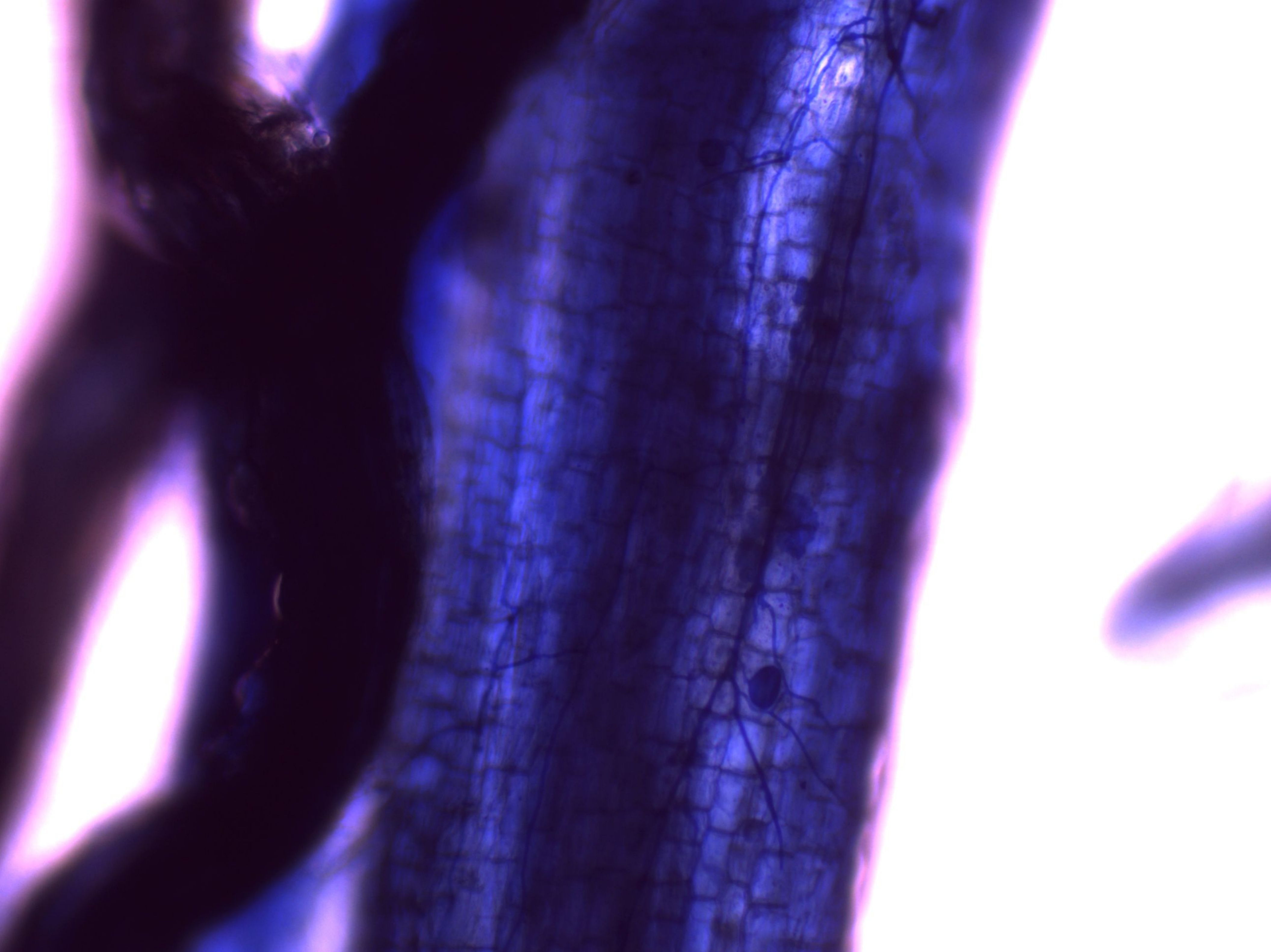

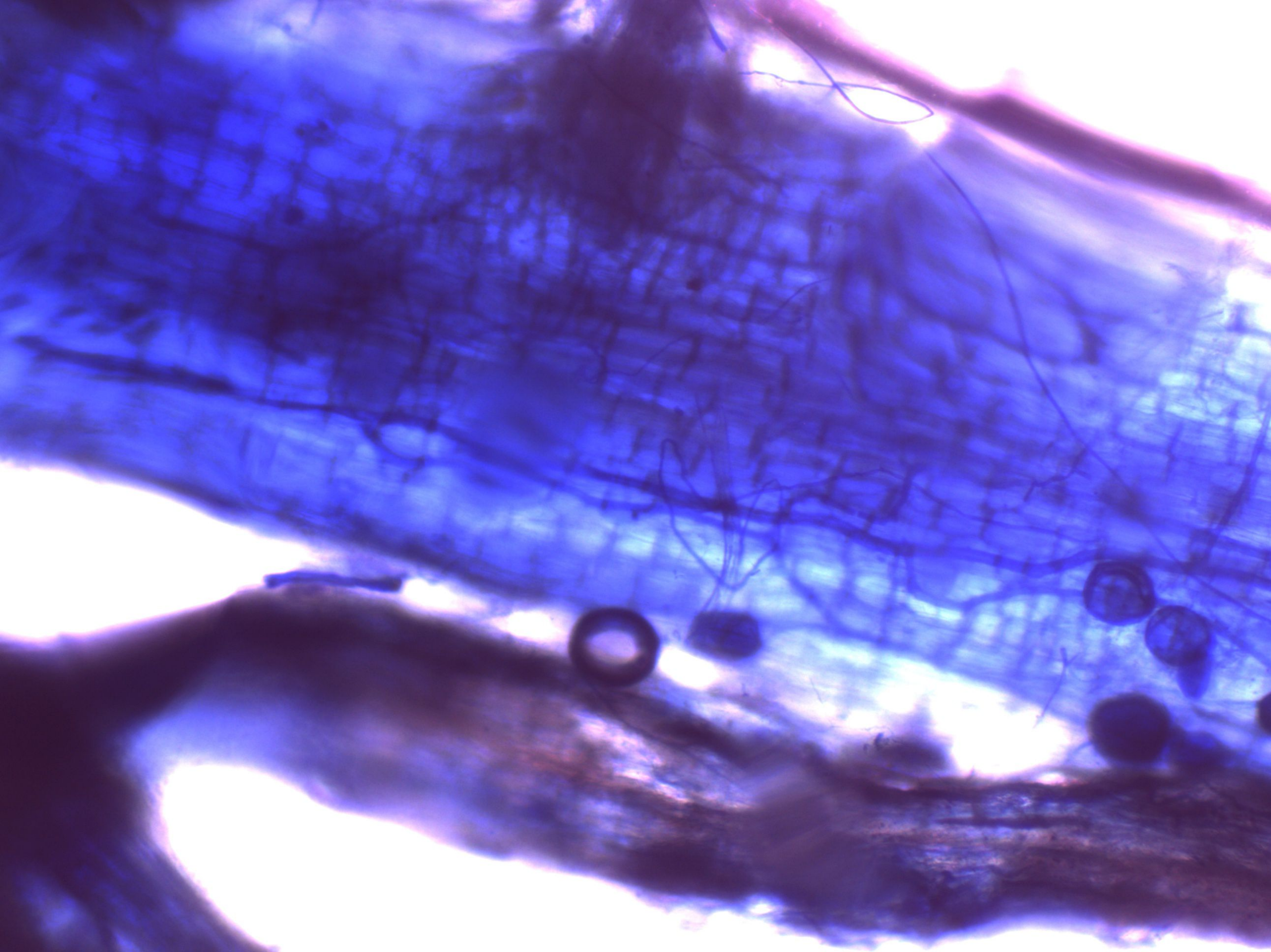

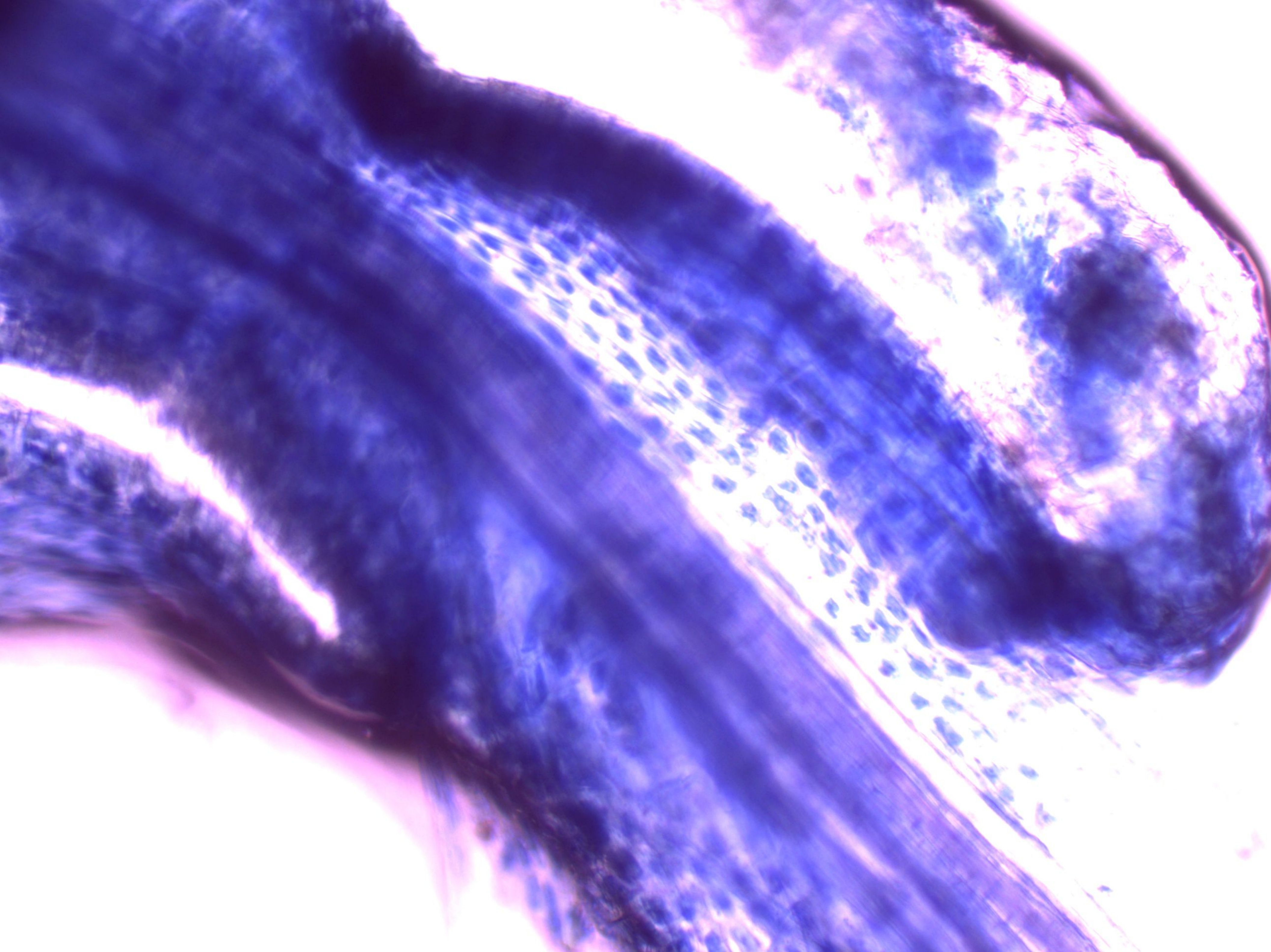

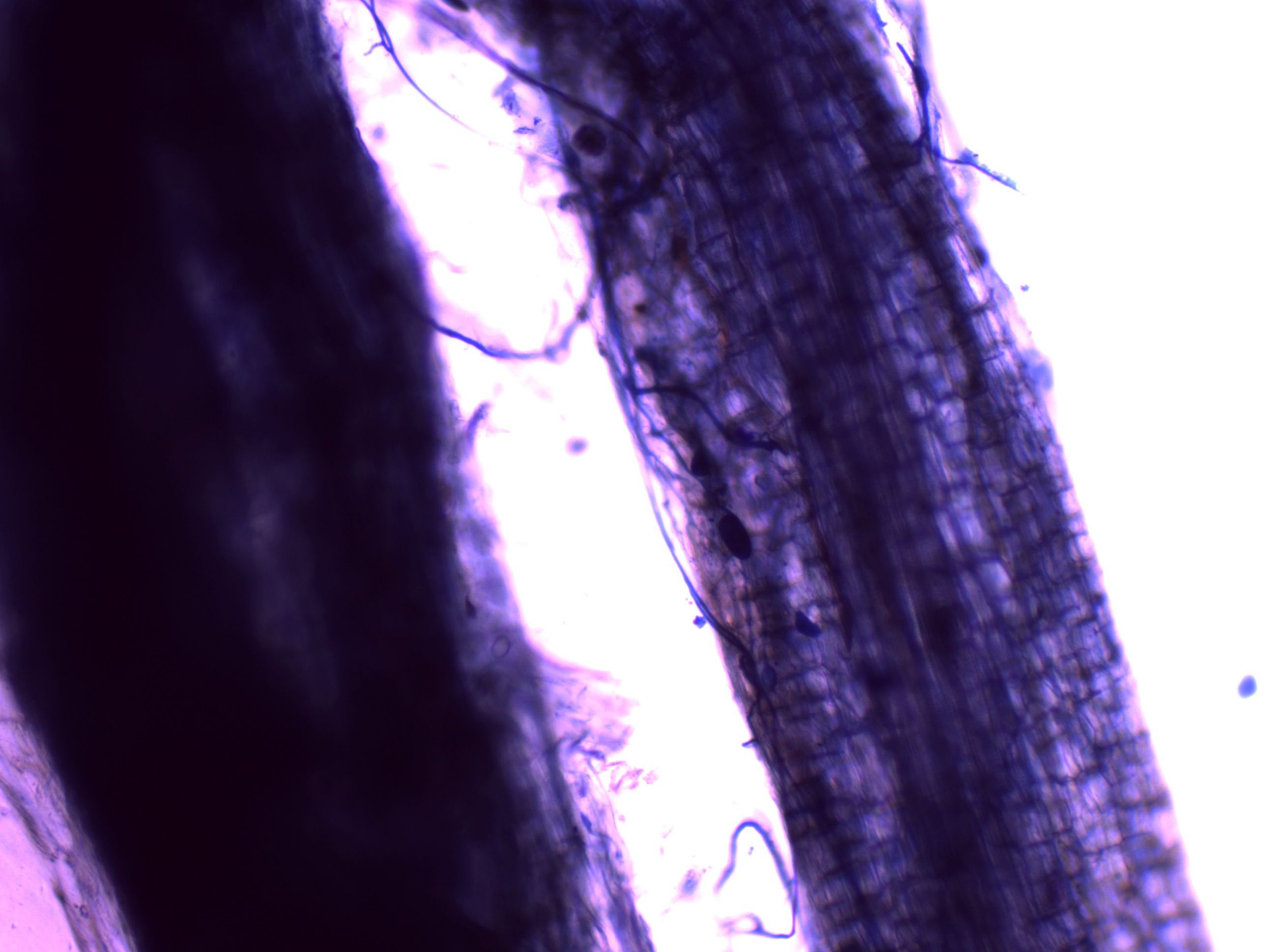

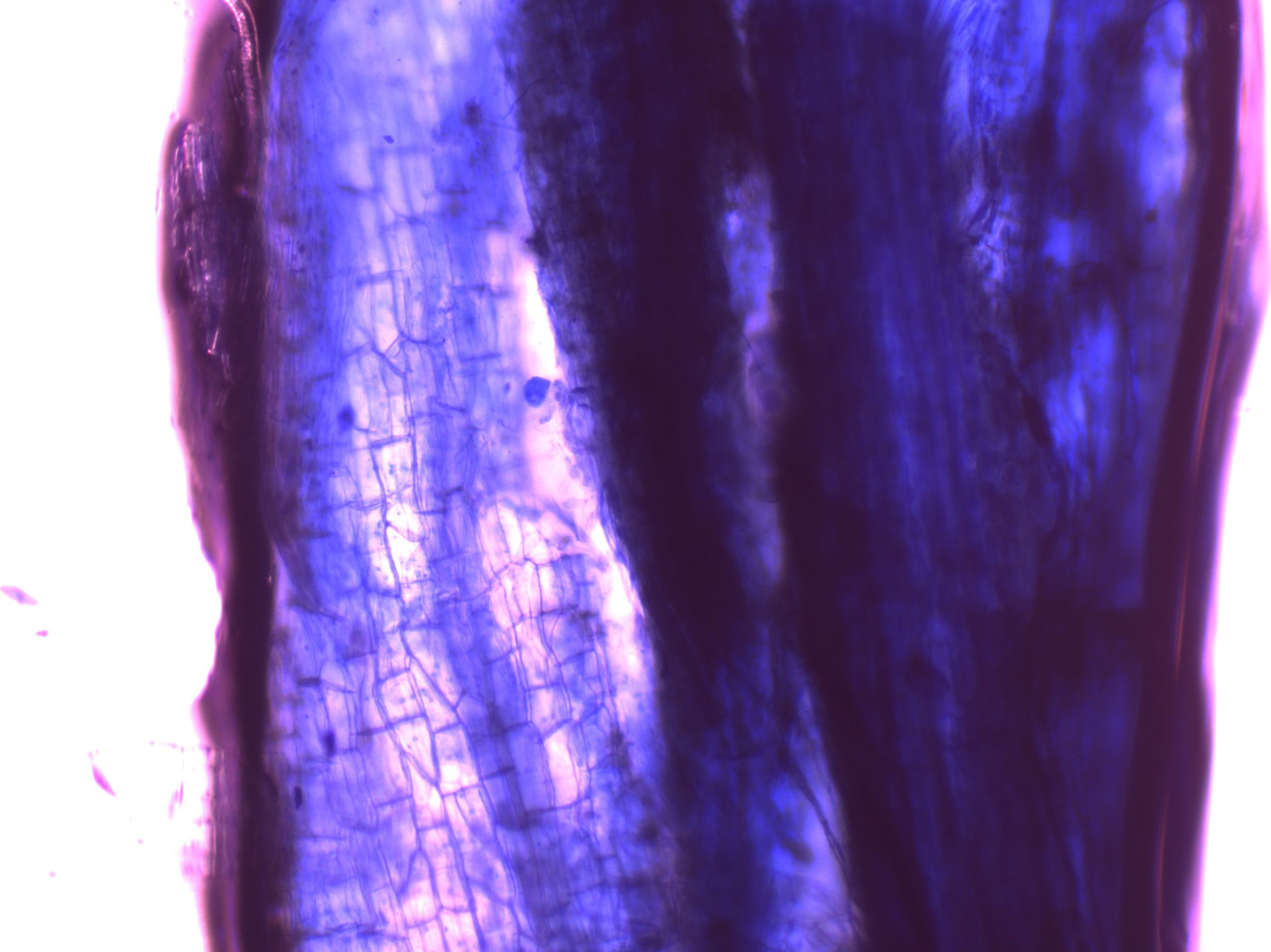

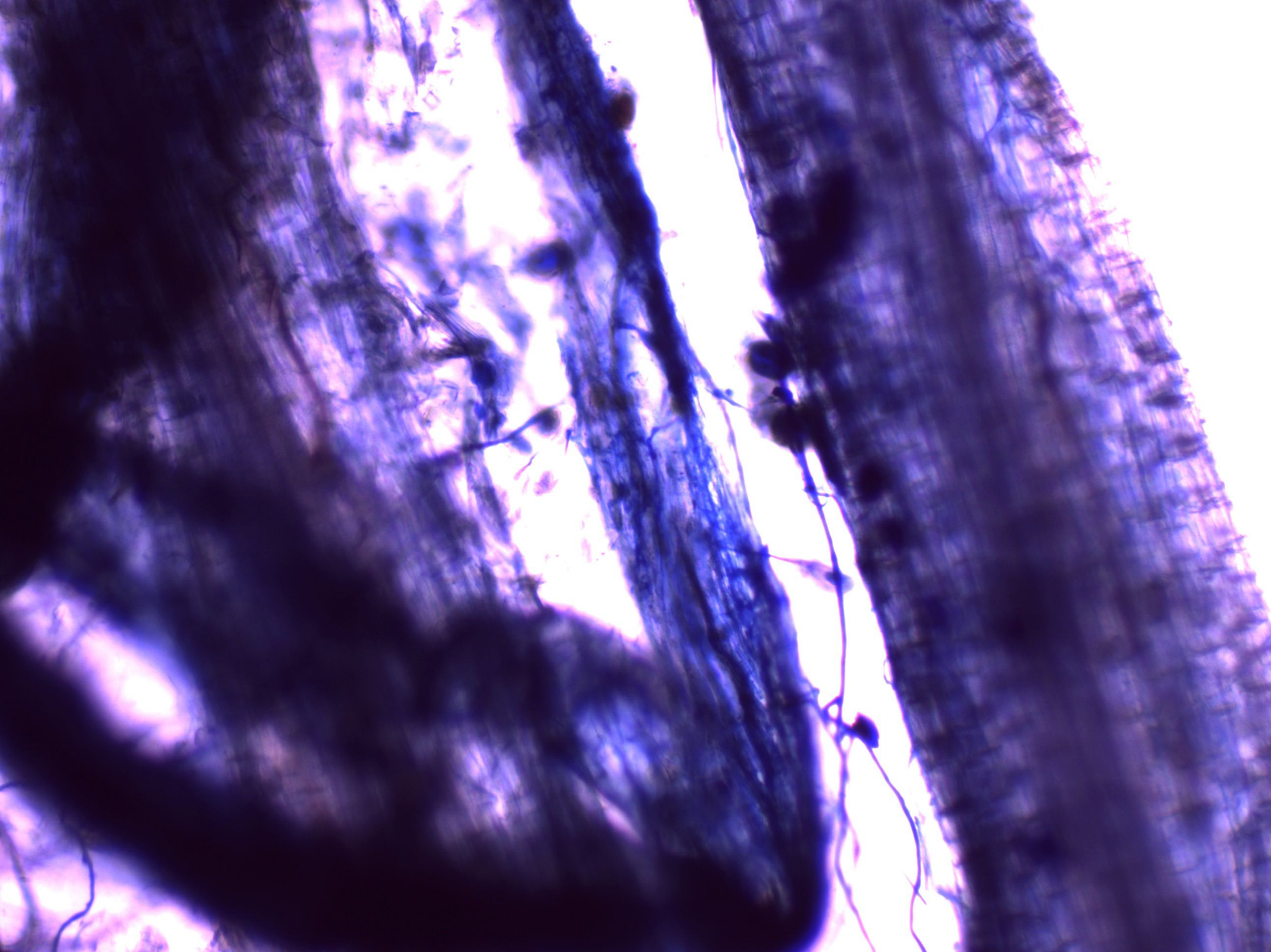

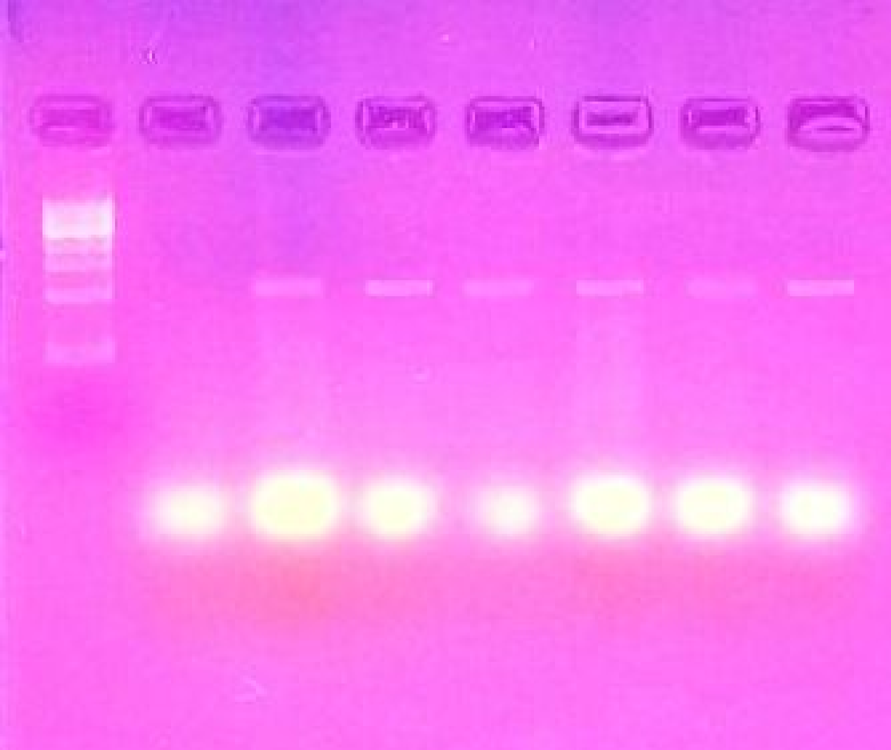

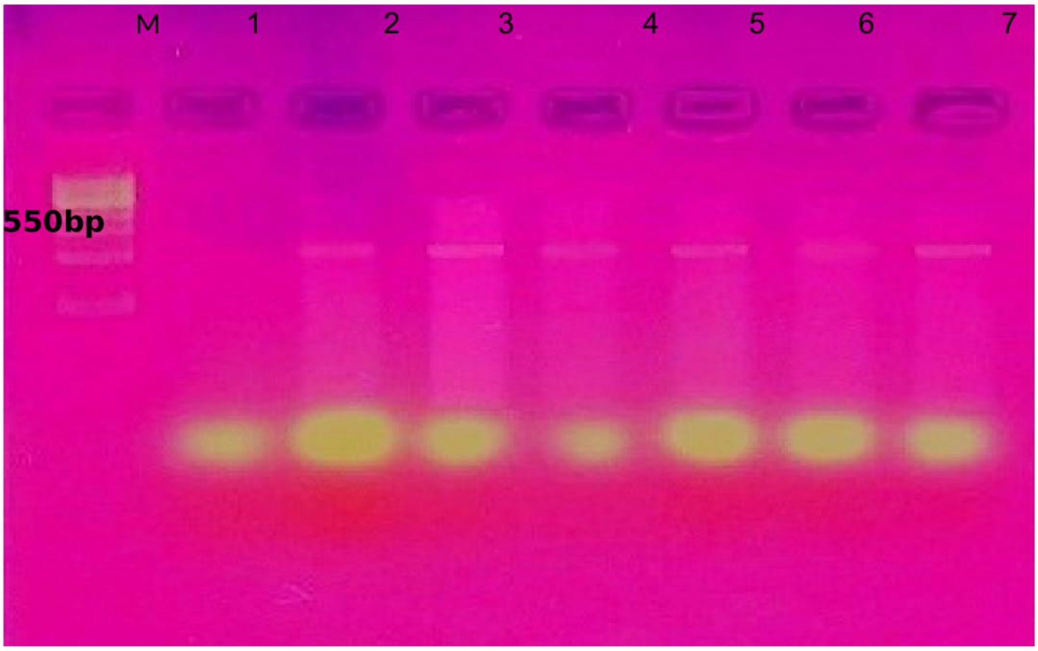

M 1 2 3 4 5 6 7
550bp

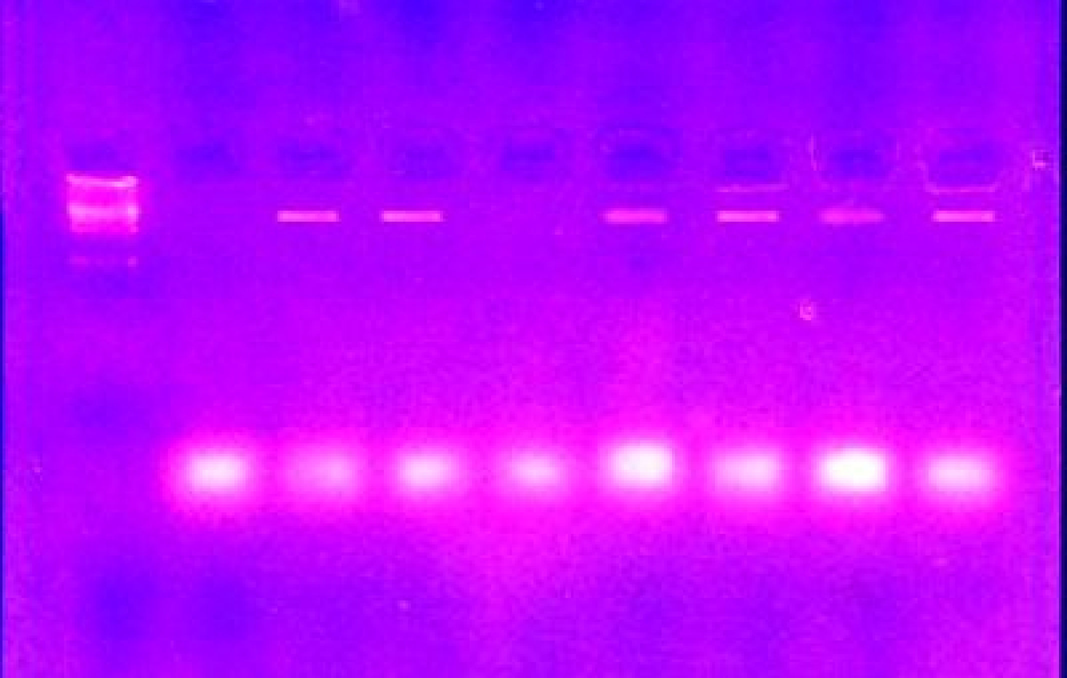

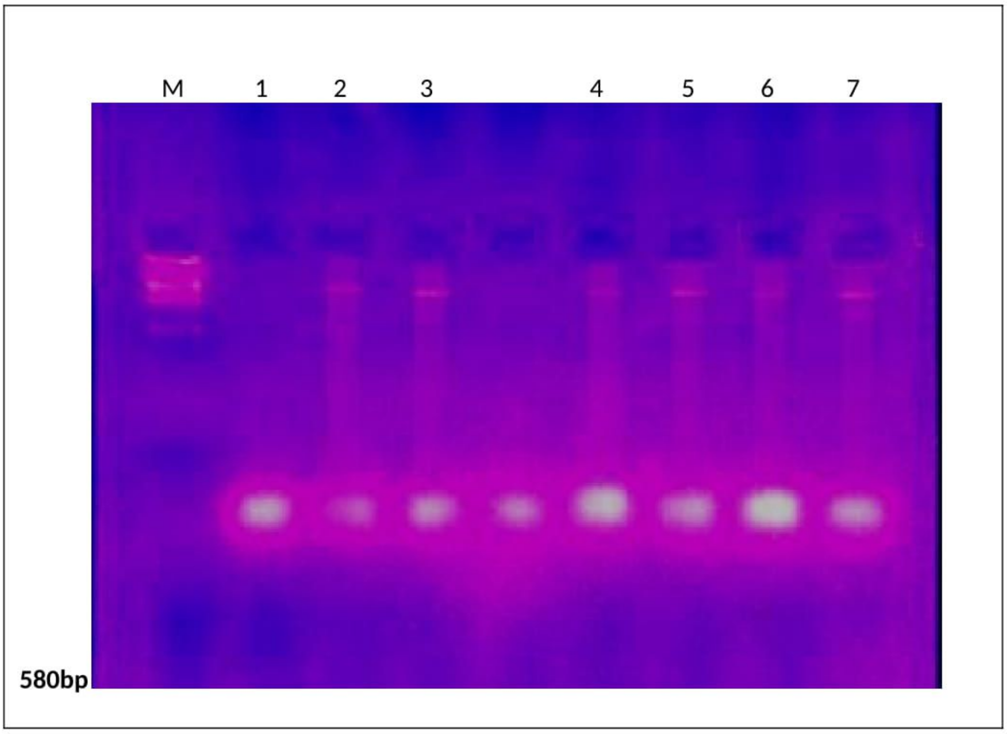
M
1
2
3
4
5
6
7
580bp

.
