# Supplementary material for: Influence of Funneliformis mosseae enhanced with titanium dioxide nanoparticles (TiO2NPs) on Phaseolus vulgaris L. under salinity stress
Source: PLoS One. 2020 Aug 20;15(8):e0235355. doi: 10.1371/journal.pone.0235355 (PMC7446817; doi:10.1371/journal.pone.0235355)
Supplement: Attachment — Submitted filename: Responce to Reviewers.docx [file pone.0235355.s005.docx]

**Responses to reviewers**

I would like to thank the reviewers

**Reviewer 1**

To change the title by e.g., “Influence of Funneliformis mosseae enhanced with titanium dioxide nanoparticles on Phaseolus vulgaris L under salinity stress”, since nowadays Glomus mosseae is classified as Funneliformis mosseae and it also can increase the interest of the potential readers;

I thank the reviewer by providing us with the recent update about the taxonomic issues of mycorrhiza. The correct and the name given by the reviewer is provided in the text.

Delete abbreviation of short expression, use full words. Abbreviations typically decrease the number of readers because is interdisciplinary;

We followed the instruction for authors as can as possible. The acronyms are deleted with exception of some mandatory one.

Some keywords do not reflect the content of the manuscript. I suggest using the following keywords:
Glomeromycota instead Microbiology and Mycology;
Funneliformis mosseae instead Glomus mosseae
Common bean roots instead Plant science
Activity of arbuscular mycorrhizal fungi instead life science and environmental sciences
Nanotechnology

Salinity stress

The keywords suggested are given and really are more understanding.

In the introduction, please make it more clearly what is the novelty (scientific/technical) of your work, approach, and results compared to already well-known facts. Most facts and trends given in the text of this article are much too vague. All trends, facts, and results must be supported by precise terms and data/numbers;

This point is very important and we thank the reviewer. The introduction was written again taking in account the last published perspectives in this field and the reviewer’s comments become more understanding.

Please, to provide clear hypotheses based in more than two scientific statements;

L53-54 I can’t see the linkage to the above paragraphs;

Introduction now is a bulk unit containing a sequence of thoughts and shows the need to do such work.

L66 Funneliformis mosseae instead Glomus mosseae;

It was changed within the whole manuscript

L69 The description of the “source of mycorrhiza, pot and potting mixture” section is not clear. I suggest it be made clearer to facilitate the evaluation of the MS;

The providing of the stock soil containing AFM was showed as it was part of our previous work. The stimulation of AFM growth and preparation of inocula are given in the text.

L82 Use “chemical analysis” instead “biochemical analysis;

This note is considered and corrected in the text

L83.soilpH?

pH at (7.9) and present within the manuscript

L85-86 The pots and soil were sterilized?

The soil was sterilized by autoclave and its details are given in the text. The pots were sterilized by their keeping in UV chamber for 12 h

L107 How many blocks?

6 replicates were allowed for each treatment. About 12 treatments as given in Table (1) were carried out

L108-113 The description of the treatments is not clear. I suggest to use a table to facilitate the reader to see the differences among the studied treatments;

I thank the reviewer for such comment. Treatments are given in Table (1). 6 replicates from each treatment were carried out.

L108, 109, 110, 111, 112, 113 Funneliformis mosseae instead Glomus mosseae;

It was corrected in the whole manuscript.

L118 To many dissimilar variables into the same subsection. I suggest split it in four subsections: 1) AMF (e.g., Colonization and mycorrhizal dependency); 2) Plant biomass and protein; 3) A&A P activity; and 4) Plant macronutrient contents. Also, the authors must describe all methods clearer;

This is good comment and subsections were made according to the reviewer comment and such brain storm really improved the manuscript.

L21, 118 Use the word “biomass” instead “mass” or even “matter” to describe plant/shoot/root dry biomass;

It was changed in the manuscript

L133 Since AMF are classified into the Phylum Glomeromycota, I suggest using only the word “spores” instead “Chlamydospores”;

This is corrected in the whole manuscript and pointed by yellow colour

L191 I expected to see a principal component analysis or even a non-metric multidimensional scaling to describe the similarities/dissimilarities among the treatments considering all matrix;

I thank the reviewer for such comment. The principal component analysis and a non-metric multidimensional scaling already made among the treatments considering all matrix with total Variance and Component	Matrix^a^	and inserted as (Figure 8 a, b) in Figure file also explained in result section (last paragraph) by yellow colour. Principal component analysis (PCA) was performed on measured parameters of common bean plants under salinity stress in response to treatments with AMF and TiO_2_NPs (Fig. 8A and B). Interpretation of the principal components (PCs) was aided by inspection of the factor-loading matrix extracted from a Varimax rotation with Kaiser Normalization of main components to identify factors responsible for the grouping of the dataset. As shown from scree plot graph of eigenvalues and loadings plot (Fig. 8A and B), the first three components; F %, M % and A % have higher eigenvalues than other measured components. Therefore, the two components were extracted describing approximately 88.81%. Accordingly, principal component analysis gave evidence to the importance of parameters *i.e.*M (%) and A (%), especially F (%), as it was considered a good and more reliable indicator for development of AMF in the presence and absence of TiO_2_NPs under salinity stress.

The presentation of the results can be improved using multivariate analysis.

Variance ANOVA analysis was done and showed the variance and significance or not. Results were taken as means and there are showed as a, b, c, d in tables; each with certain weight; LSD: At significant level (P> 0.05).Sample symbols (a.a) mean non significant difference (a.b) mean significant difference

To many tables. I suggest presenting only significance results. The results without significance differences must be added as an electronic supplementary material;

Both Table 4 and 7 as labeled in un revised manuscript , that without mandatory significance were changed to supplementary material **Supplementary Table 1.** Effect of salinity levels on sodium (Na), calcium (Ca), magnesium (Mg) and translocation.

**Supplementary Table 2 .** Amplified Nest and chitin synthase Quantity (Intensity & Relative density) of mycorrhiza levels of Phaseolus plants inoculated with ***Funneliformis mosseae*** and TiO_2_NPs in the presence of salinity.

The discussion needs to be improved by explaining further the results and the mechanisms underlying salinity, the use of nanoparticles and the growth of inoculated plants with AMF;

These concepts and explanations are given in the text.

**Reviewer #2:**

1. The title should be revised by adding “(TiO2NPs)” particularly after titanium dioxide.
It is done and mentioned in the title

2. Line 21: Add “, respectively” after salinity level.
It is done and mentioned in the title

3. The introduction is nicely written and the problem is discussed well.
Thank you very much for this point. Introduction is rewritten and discussed again.

4. The authors should add some reports based on the use of NPs for enhancing the soil-plant interactions to strengthen the proposed hypothesis in the introduction section.

Thank you very much for this point. Introduction was rewritten again taking in account the reviewer remarks.

5. Line 61: Thus, ....., the line should be started from a new paragraph. It will bring clarity between the novelty and objectives of the study.
This error is corrected

6. Line 74: Describe why the authors selected the particular local strain of mycorrhiza with its industrial possibilities.

This was our isolate and was used by our Lab in a previous work (Rabie et al., 2005).

7. Line 75: The authors should provide a brief methodology for this section. The data may be provided as supporting information files to avoid text plagiarism, if.

This is given in details and become more understanding.

8. Line 80: Provide geocoordinates of Sharkia province.
The geocoordinates of Sharkia province is provided within the manuscript. It follows Egypt.

9. Most of the methodologies are just mentioned in short sentences which are not recommended. Provide brief protocols for them.
Thank you for this comment. However, certain length of manuscript is allowed for each manuscript. Some of them are given as supplementary file.

10. Line 85: Why you have used an abbreviated form of Dept. Agric. Res. Centre, Giza, Egypt?
This abbreviation is changed to the whole word

11. Line 92: add space between TiO2NPswere.
It is done.

12. Line 98: Direct use of numerical value in the starting of paragraph and sentence? Why?
Such mistakes are corrected as each sentence contained subject +verb+ the rest of the sentence. Sorry.

13. Check for potential typing errors “breaks, spaces, dots, etc., throughout the manuscript.
The grammar and the whole manuscript are reviewed again by Prof. Dr. G. Enan.

14. Line 191: Provide names of software used in this study.
The software is added in the manuscript (WASP software version 2.0).

15. Line 197: Better to provide the information of the FTIR spectrum (9 peaks) as a single table data.
The information of the FTIR spectrum (9 peaks) is already presented in a single table 2.

16. Some asterisks given in the table data are not defined in their footer captions.
These points are very important; the corrections were made and explained.

17. Use uniform font and text formatting in the manuscript.
It was done within the whole manuscript.

18. The level of statistical significance should be provided in the manuscript wherever it is mentioned.
Thank you very much for this point. The level of statistical significance is showed in the manuscript.

19. Have you observed some negative effects of low NP treatment and high salt concentration? Describe in discussion section accordingly with supportive literature.
This is done in the manuscript and some paragraphs are inserted in the discussion section and pointed by green colour.

20. Many references are outdated. Authors are required to replace them with the latest one including https://doi.org/10.1016/j.bcab.2019.101463.

As update of references were made and recent ones were added.

21. Figs. resolution is too low and difficult to understand.

The quality of the figures improved.
